# Effect of childhood maltreatment on cognitive function and its relationship with personality development and social coping style in major depression disorder patients: A latent class model and network analysis

**DOI:** 10.3389/fpsyt.2023.748857

**Published:** 2023-01-20

**Authors:** Xiao Wang, Xinrong Li, Juan Zhao, Xinzhe Du, Junxia Li, Wentao Zhao, Jing Li, Sha Liu, Yong Xu

**Affiliations:** ^1^Shanxi Key Laboratory of Artificial Intelligence Assisted Diagnosis and Treatment for Mental Disorder, First Hospital of Shanxi Medical University, Taiyuan, Shanxi, China; ^2^Department of Psychiatry, First Hospital/First Clinical Medical College of Shanxi Medical University, Taiyuan, Shanxi, China; ^3^Department of Psychiatry, Taiyuan Central Hospital of Shanxi Medical University, Taiyuan, Shanxi, China

**Keywords:** childhood maltreatment, personality characters, cognitive function, latent class analysis, network analysis

## Abstract

**Study objectives:**

The study aimed to (1) analyze the interrelationships among different types of childhood adversity, diverse personality dimensions, and individual coping style integratively among major depressive disorder (MDD) patients and healthy participants using a network approach; (2) explore the latent class of child maltreatment (CM) and its relationship with cognitive function.

**Methods:**

Data were collected from the Objective Diagnostic Markers and Personalized Intervention in MDD Patients (ODMPIM) study, including 1,629 Chinese participants. Using the Childhood Trauma Questionnaire to assess CM, the Simplified Coping Style Questionnaire to measure individual coping style, Eysenck Personality Questionnaire Revised-Short Form for personality characters, and a series of neurocognitive tests, including seven tests with 18 subtests for cognitive assessments. We used the “Network Module” in Jeffreys’s Amazing Statistics Program (JASP) and R package for network analysis. A latent class analysis was performed with SAS9.4.

**Results:**

Child maltreatment was more common in MDD patients than in healthy controls, except for emotional abuse. Network analysis showed that emotional abuse, emotional neglect, physical abuse, and physical neglect formed quadrangle connections. Personality dimensions were associated with physical neglect and emotional abuse. All types of CM (excluding sex abuse) showed an association with coping style. Emotional neglect showed the highest centrality measures. Physical neglect had a high level of closeness. To a concerning strength, emotional and physical neglect showed the highest levels. The structure of the networks is variant between groups (M = 0.28, *P* = 0.04). Latent class analysis (LCA) revealed that three classes provided the best fit statistics. Neglect and abuse classes tended to perform more poorly on the five cognitive domains.

**Conclusion:**

This study provided insights on multi-type of CM. Neglect played an important role in different routes through the relation between CM with personality traits and social coping style. However, neglect has often been ignored in previous studies and should receive more public attention.

## 1. Introduction

Child maltreatment (CM), including abuse (physical, emotional, and sexual) and neglect (physical and emotional) of young people under the age of 18, is a sensitive and complex issue in terms of both clinical practice and research (1). It’s the single most influential known cause of lifetime mental health impairment that is preventable (2). The World Health Organization (WHO) has defined CM as all forms of physical and emotional ill-treatment, sexual abuse, neglect, and exploitation that results in actual or potential harm to the child’s health, development, or dignity (3). The conservative estimate of CM’s prevalence is approximately 15% in high-income countries (4, 5), and the global prevalence of CM is approximately 50% (3).

The consequences of CM across the lifespan have been well-documented in the literature. CM has been shown to have a causal relationship or be associated with a number of mental and physical health problems, such as personality disorders, depression, anxiety, and diabetes (6–8). Most of the studies considered diseases rather than other psychosocial development (such as personality traits, cognitive function, and negative attitude) as primary outcomes. In general, previous research has documented a negative association between CM and academic performance (9, 10), with maltreated children being more likely to have lower average academic, educational levels, and employment rates. This might be caused by the poor executive functioning of children who have experienced maltreatment, leaving them with limited cognitive capacity to devote to learning tasks. However, relatively little research has been conducted on the relationship between CM and cognitive function.

Most studies view CM as a whole (11). However, some suggest that different types of CM have distinct effects on personality dimensions. Hengartner et al.’s study (12), conducted as part of the Epidemiology Survey of the Zurich Programme for Sustainable Development of Mental Health Services, showed that emotional abuse had the most substantial effects on neuroticism, openness, conscientiousness, extraversion, and agreeableness. Physical abuse was associated with higher neuroticism, higher openness, and lower agreeableness, while sexual abuse was only related to higher neuroticism. Few studies have explored the relationships between different types of CM and various personality dimensions integratively (13). The fact remains that CM types are intercorrelated, and this needs to be considered when interpreting results. Such strongly interconnected relationships between adversity and personality dimensions require more integrated analysis beyond testing one-to-one relationships. Network analysis makes it possible to study such complex multidimensional relationships integratively. It can be a powerful tool to explore multicollinearity and predictive mediation and can even be used to highlight the presence of latent variables (14, 15).

Moreover, most researches have examined the general population. However, CM is more prevalent among patients with depression. We all know that CM increases the risk of depression, but not all abused children become depressed. Some factors increase vulnerability to or act as a buffer against depression. It has been suggested that coping styles and personality traits are involved in this pathway (16). Coping styles can be divided into positive coping and negative coping. Positive coping refers to taking a direct and rational approach to solve a problem, while negative coping refers to dealing with issues by avoidance, withdrawal, and wishful thinking (17). The evidence indicates that the tendency to use specific types of coping styles is likely to arise from CM and that these styles are associated with depressive symptoms (18). Personality traits are also associated with depression (19), while the current results of the specific personality traits linking with CM and depression seem inconsistent (20). They also influence individuals’ strategies for coping with anxiety and depression. So there might be different networks of these factors between depression patients and healthy people (21), and that might constitute one of the pathological mechanisms of depression.

According to findings from prior studies, we hypothesized that latent classes of CM in major depressive disorder (MDD) patients and healthy controls (HC) group could be identified based on their historical experiences with CM types. For example, there would be at least one latent class with multiple types of CM, and cognitive function would vary among different classes. Furthermore, we hypothesized that different types of CM such as sexual abuse, other abuse, and neglect would be significantly and uniquely associated with many dimensions of personality pathology and coping style and the networks differed between these two groups. We performed a study in a sample of patients with MDD and healthy controls, aiming to (1) analyze the interrelationships among different types of childhood adversity, diverse personality dimensions, and individual coping style integratively among MDD patients and healthy participants using a network approach; (2) explore the latent class of CM and its relationship with cognitive function.

## 2. Materials and methods

### 2.1. Study design and participants

Data were collected from the Objective Diagnostic Markers and Personalized Intervention in MDD Patients (ODMPIM) study, which was carried out between December 2013 and December 2016. The participants were part of a clinical trial and a detailed description of the ODMPIM study protocol can be found elsewhere (22). Briefly, the participants were recruited from nine clinical sites at nine top tertiary hospitals (seven within academic settings and two in clinical practices) located in six cities. The number of cases recruited by each clinical site is shown in Supplementary Table 1. The details of inclusion and exclusion criteria for each group are listed in Supplementary Table 2. Finally, this study included 1,629 Chinese participants (1,130 patients with a current diagnosis of MDD and 499 healthy controls) aged 18–55 years. The validated Chinese version of the Mini-International Neuropsychiatric Interview (MINI, Version 5.0) was used to diagnose current MDD. The 17-item Hamilton Depression Rating Scale (HAMD-17) was applied to assess the severity of depression over the past 2 weeks. The study was approved by the Ethics Committee of Peking University Sixth Hospital (Approval No. 2013-29-1). Before respondents were interviewed, written informed consent was obtained from them.

### 2.2. Assessment

All assessments were acquired at baseline prior to randomized treatment in the ODMPIM study. The measurement scales included the Childhood Trauma Questionnaire (CTQ), the Simplified Coping Style Questionnaire (SCSQ), and a series of neurocognitive tests.

#### 2.2.1. CM

The CTQ (23) was used to assess retrospective, self-reported experiences of five domains of CM, including emotional abuse (EA), physical abuse (PA), sexual abuse (SA), emotional neglect (EN), and physical neglect (PN). EA was defined as, “verbal assaults on a child’s sense of worth or well-being or any humiliating or demeaning behavior directed toward a child by an adult or older person.” PA was defined as, “bodily assaults on a child by an adult or older person that posed a risk of or resulted in injury.” SA was defined as “sexual contact or conduct between a child younger than 18 years of age and an adult or older person.” EN was defined as, “the failure of caretakers to meet children’s basic emotional and psychological needs, including love, belonging, nurturance, and support.” PN was defined as, “the failure of caretakers to provide for a child’s basic physical needs, including food, shelter, clothing, safety, and health care” (23). There are 28 items in the CTQ, with each dimension containing five items scored on a five-point Likert-type scale ranging from “never true” to “very often true.” The last three items are regarded as validity evaluation questions. According to the retrospective self-assessment questionnaire manual by Bernstein et al., participants meeting the criteria for each particular domain (EA > 12 or PA > 9 or SA > 7 or EN > 14 or PN > 9) were considered “exposed” to clinically significant levels of CM, while those not meeting these criteria were regarded as “non-exposed” (24, 25).

#### 2.2.2. Coping style

The SCSQ is a 20-item self-report scale measuring individual coping style with two subscales: positive coping (12 items) and negative coping (eight items) (26). Positive coping reflects the level of the active coping style, such as “when facing problems, finding several different solutions.” In contrast, negative coping reflects the level of passive coping style, such as “when facing problems, escaping troubles by drinking and smoking.” Each item is scored on a four-point Likert scale ranging from “never” to “always.” The total scores on each subscale reflect the level of the coping style.

#### 2.2.3. Personality characters

Eysenck Personality Questionnaire Revised-Short Form (EPQR-Short) (27) is a self-reported questionnaire with 48 items. The questionnaire covers four domains: neuroticism (N), extraversion (E), psychoticism (P), and the lie scale (L), with 12 items for each domain. Each question has a binary “yes” or “no” response and is scored 1 or 0. Total scores on each subscale reflect the level of the scale.

#### 2.2.4. Cognitive assessments

Cognitive assessments were performed at admission by trained investigators using a series of neurocognitive tests, including seven tests with 18 subtests. These tests covered five cognitive domains (28): Attention/vigilance was assessed with the Continuous Performance Test-Identical Pairs. For learning and memory, the immediate recall and delayed recall sections of the Hopkins Verbal Learning Test-Revised and the Brief Visuospatial Memory Test-Revised were used. Processing speed was assessed by the Color Trails Test I. The Stroop Color-Word Test (word task and color task), the Digit-Symbol Coding Test, the Color Trails Test II, the Stroop Color-Word Test (word interference task), and the Animal Verbal Fluency Scale were used to assess executive function (29). The Continuous Performance Test-Identical Pairs test was carried out on a standardized computerized screen while the other tests were performed using standardized scales. The raw score of each test could be calculated for further analysis.

### 2.3. Statistical analyses

After systematic data cleaning, the categorical variables were presented as frequencies and percentages. For the continuous variables, a Kolmogorov–Smirnov (K-S) test of normality was performed, and the data were expressed as the mean (standard deviation) if they were normally distributed and the median (interquartile range) if they were not. An independent-sample t-test, Mann–Whitney *U* test, Chi-square test, and Fisher’s exact test were used to compare the demographic data between MDD and HC groups, as appropriate. The differences in CM, personality traits, and coping styles between the two groups were tested by logistic regression and linear regression controlled by age, sex, and education levels. Significance levels was considered pre-set to 0.05 based on two-tailed tests.

#### 2.3.1. Network relationship

Network analysis has been increasingly used for exploratory studies of psychological behavior. This differs from the traditional perspective where latent variables are thought to explain the correlation among variables, and observed variables are assumed to influence one another causally. The network is established based on partial correlations between variables. A partial correlation network makes it possible to identify unique interactions between variables that cannot be identified using multiple regression analysis (30). In this study, 11 nodes/symptoms were included: (1) five subscales of CM as measured by the CTQ, (2) four domains of personality traits as measured by EPQR-Short, (3) and two coping styles as measured by the SCSQ. Pearson correlation analyses were used to estimate strengths of association between nodes, with thicker edges indicating stronger relationships. Network models from the two groups were estimated separately using sparse Graphical Gaussian Models (GGM) combined with a graphical least absolute shrinkage and selection operator (LASSO) method (31); model selection was based on the Extended Bayesian Information Criterion (EBIC) (32). To conduct the network analysis, we used the Network Module in Jeffreys’s Amazing Statistics Program (JASP) (version 0.9.0.1), which uses the R package qgraph with “EBICglasso” estimation (33).

Three centrality measures were applied to investigate the importance of each node: betweenness, closeness, and strength (34). Betweenness equals the number of shortest paths between every combination of two nodes that pass through the node of interest. Nodes with a high betweenness centrality score are the ones that most frequently act as “bridges” between other nodes, which indicates the importance of relationships with other nodes. Closeness helps find the nodes closest to the other nodes in a network based on their ability to reach them. High closeness indicates a short average distance to all other nodes. Strength measures the sum of absolute edge weights of all direct connections between a specific node and other nodes. It helps to find the nodes with the highest number of links to other nodes in the network. Robustness analyses were conducted using the R-package “bootnet” (35). Non-parametric bootstrapping (1,000 replicates) was performed to estimate 95% confidence intervals (CI) of edge values. To clarify the replicability of the edge weights and the centrality measures, robustness coefficients (randomly dropping 10, 20, …, 90% participants from the sample and recomputed centrality estimates) for centrality measures were calculated. Finally, the differences in the estimated network between these two groups were tested by the R package “NetworkComparisonTest (NCT)” (36) based on several invariance measures, including network structure, edge strength, and global network strength.

#### 2.3.2. Latent class of CM

Latent class analysis (LCA) is a statistical technique used in factor, cluster, and regression techniques. It is a subset of structural equation modeling (SEM) for binary variables. In this study, LCA was performed on the five subscales of CTQ with SAS 9.4. To find the best solution, classes need to be decided when they are as homogeneous as possible, and differences between them should be as significant as possible. We evaluated models ranging from two to five classes. The following statistics were used to evaluate model fit: Akaike’s Information Criterion (AIC), Bayesian Information Criterion (BIC), the Sample-Size Adjusted Bayesian Information Criterion (SS-BIC), entropy, the Lo-Mendell-Rubin likelihood ratio test (LMR-LRT), and the bootstrapped likelihood ratio test (BLRT). Lower values of the BIC, SS-BIC, and AIC, and higher values of entropy indicate better fit. A significant LMR-LRT or BLRT suggests that a model fits the data better than a model with one less class (37).

#### 2.3.3. Correlation between latent classes of CM and cognitive function

After adjusting by age, sex, and years of education based on Chinese norms (28), the raw score of each test was converted to a T-score. A higher score indicated better performance. The score of each cognitive domain was obtained by averaging the scores of the tests comprising the domain. One-way ANOVA was used to determine the significance of the differences among latent classes. Significance levels for all statistical tests were set to *P* = 0.05. If there were significant differences, *post hoc* multiple comparisons were conducted using the Student–Newman–Keuls (SNK) test.

## 3. Results

### 3.1. Sample description

In total, 1,130 patients with MDD and 499 healthy controls participated in this study. Twenty-one patients with incomplete demographic data (age, work status, marriage, and education) were excluded. Finally, 1,608 participants were included in this study. 53.8% of MDD patients were assessed as having moderate depression by HAMD-17, and the median score was 21.0 (17.5, 24.0). The median and interquartile range of illness duration were 6.0 (3.0, 13.0) months. The daily antidepressant doses were converted to fluoxetine equivalent doses using the following equations: fluoxetine 40 mg/d = citalopram 40 mg/d = escitalopram 18 mg/d = sertraline 98.5 mg/d = paroxetine 34 mg/d = fluvoxamine 143.3 mg/d. The median and interquartile range of the daily dosages after converting to fluoxetine equivalent doses was 23.53 (20.28, 33.33) mg/d. All the healthy controls had lower HAMD-17 scores, with none of them being assessed as a depressive state. The mean age of the MDD and healthy groups was 39.40 and 33.95, respectively. There were significant differences in social-demographic information between the two groups. Details are provided in Table 1.

**TABLE 1:** Social-demographic information and prevalence of CM for MDD and HC group.

		MDD		HC		*P*
		***n***	**%**	***n***	**%**	
Age, years	Mean (SD)	39.40 (10.76)		33.95 (9.05)		<0.001
Gender	Female	776	69.41	299	61.09	
Educational level	Primary school or below	78	6.98	17	3.47	<0.001
	Junior/senior high school	504	45.08	87	17.76	
	Undergraduate or above	536	47.94	386	78.78	
Employment	Full-time job	740	66.19	442	90.20	<0.001
	Part-time job	25	2.24	13	2.65	
	Retired	81	7.25	7	1.43	
	Unemployed	131	11.72	12	2.45	
	Housewife	141	12.61	16	3.27	
Childhood maltreatment	EA	225	20.13	103	21.02	0.6818
	PA	45	4.03	5	1.02	0.0004
	SA	121	10.82	21	4.29	<0.001
	EN	212	18.96	41	8.37	<0.001
	PN	55	4.92	9	1.84	0.0011
	CM	465	41.59	152	31.02	<0.001

EA, emotional abuse; PA, physical abuse; SA, sexual abuse; EN, emotional neglect; PN, physical neglect; CM, childhood maltreatment.

After controlling by age, sex, and education levels, CM’s prevalence rates and severity scores were higher in MDD patients than in healthy controls, except for emotional abuse. Besides, scores on all personality dimensions differed significantly between the MDD and healthy groups, except for the lie scale. The MDD group had lower positive coping style scores and higher negative coping style scores. See Table 2 for further details.

**TABLE 2:** Description of personality, coping style, and CM scale scores.

	MDD		HC		*P*
	**Mean**	**Std.**	**Mean**	**Std.**	
EA	10.46	2.46	10.69	2.21	0.0620
PA	5.71	1.88	5.37	1.05	<0.001
EN	6.78	5.29	4.61	4.68	<0.001
PN	6.62	3.23	5.21	2.58	<0.001
SA	5.33	1.09	5.16	0.64	0.0033
Positive coping style	1.36	0.58	2.00	0.53	<0.001
Negative coping style	1.19	0.56	1.12	0.54	0.0253
N	4.21	3.18	8.26	3.06	<0.001
L	5.20	2.76	5.18	2.86	0.8832
P	8.86	1.84	9.58	1.79	<0.001
E	5.89	3.25	3.34	2.69	<0.001

Std., standard deviation; EA, emotional abuse; PA, physical abuse; SA, sexual abuse; EN, emotional neglect; PN, physical neglect; CM, childhood maltreatment; N, neuroticism; L, lie scale; P, psychoticism; E, extraversion.

### 3.2. Network analysis

**FIGURE 1:**
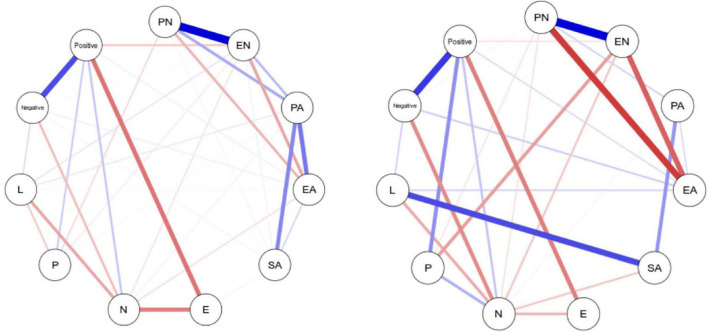
Network plot of the seven dimensions of the five types of CM, personality characters, and the scores for coping style (EN, emotional neglect; EA, emotional abuse; PN, physical neglect; PA, physical abuse; SA, sex abuse; N, neuroticism; E, extraversion; P, psychoticism; L, lie scale; Positive, positive coping; Negative, negative coping). Edges represent regularized partial correlations, where thicker edges represent stronger connections. Blue edges represent positive correlations, whereas red edges represent negative correlations.

For the relationships among different CM types, emotional abuse, emotional neglect, physical abuse, and physical neglect formed quadrangle connections in both the HC group and the MDD group. Personality dimensions showed associations with physical neglect, emotional abuse, and emotional neglect. All types of CM (excluding SA) showed an association with coping style (Figure 1). The most substantial edges were between SA and L (0.343), EN and P (–0.206), PN and negative (0.158), and EN and N (–0.149) among the healthy control group and between EN and positive (–0.103), PN and P (–0.092), EN and N (–0.081), and EA and E (0.069) among the MDD group. Bootstrapped confidence intervals for the edges were shown in Supplementary Figures 1, 2.

**FIGURE 2:**
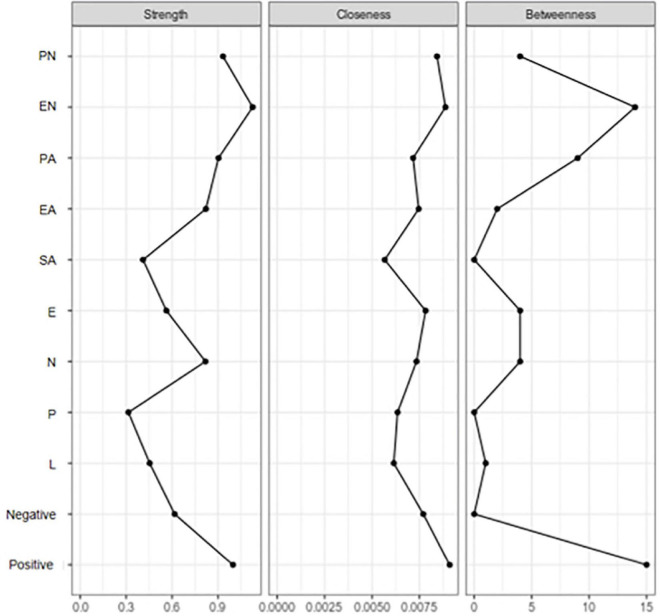
Centrality measures among MOD group (EN, emotional neglect; EA, emotional abuse; PN, physical neglect; PA, physical abuse; SA, sex abuse; N, neuroticism; E, extraversion; P, psychoticism; L, lie scale; Positive, positive coping; Negative, negative coping).

**FIGURE 3:**
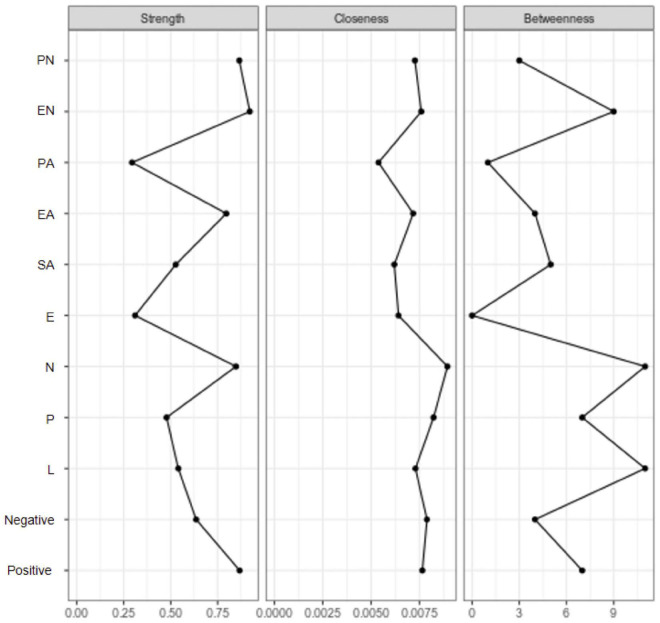
Centrality measures among healthy control group (EN, emotional neglect; EA, emotional abuse; PN, physical neglect; PA, physical abuse; SA, sex abuse; N, neuroticism; E, extraversion; P, psychoticism; L, lie scale; Positive, positive coping; Negative, negative coping).

Of all adversities, EN showed the highest centrality measures (Figures 2, 3), indicating it lay on many shortest paths between every combination of two other nodes in the network. This suggested that EN played a vital role in different routes through the network. PN had a high level of closeness, meaning it had a short average distance from all other nodes, suggesting an influential role in the network. Emotional and physical neglect showed the highest levels of strength, indicating that they had many or strong connections with other nodes in the network. Among the healthy control group, the centrality measures were much lower for neglect. PN, EN, and EA showed higher closeness and strength. Comparisons of the two networks based on NCT results showed that the structure of the networks was variant between groups (M = 0.28, *P* = 0.04), and the global network strength test similarly revealed no significant differences in the weighted sum associations of trait facets (S = 0.44, *P* = 0.41).

### 3.3. Latent class analysis

Latent class analysis revealed that three classes provided the best fit statistics. From the results of multiple-group latent class analysis, it could be seen that the latent class probabilities were different between the MDD and HC groups. Information regarding model fit statistics and selection criteria were shown in Supplementary Table 3. For the MDD group, the LMR-LRT and BLRT were significant for the two- and three-class models and marginally significant for the four-class models. The three-class solution had the lowest information criteria (AIC, BIC, and SS-BIC) and demonstrated high entropy (0.917), suggesting good class identification. Table 3 depicted the observed class membership and endorsement frequencies. Class 1 was termed “neglect,” characterized by very high endorsement rates for PN and EN. Class 2 was termed “normal.” Class 3 was termed “abuse,” characterized by prominent symptoms of EA. The proportions of these three classes were 15.63, 80.89, and 3.47%. For the HC group, the two-class solution had the lowest information criteria and demonstrated high entropy (0.920). The LMR-LRT and BLRT were all significant in two-, three-, and four-class models. The proportions of these two classes were 8.07 and 91.93%.

**TABLE 3:** Results of LCA.

	MDD			HC	
	**Neglect**	**None**	**Abuse**	**Neglect**	**None**
	15.6%	80.9%	3.5%	8.1%	91.9%
EA	0.023	0.202	0.965	0.050	0.227
PA	0.075	0.010	0.579	0.025	0.009
SA	0.051	0.039	0.265	0.0002	0.020
EN	0.474	0.025	0.359	0.563	0.0001
PN	0.928	0.029	0.551	0.856	0.020

EA, emotional abuse; PA, physical abuse; SA, sexual abuse; EN, emotional neglect; PN, physical neglect; CM, childhood maltreatment.

### 3.4. Neurocognitive tests

**FIGURE 4:**
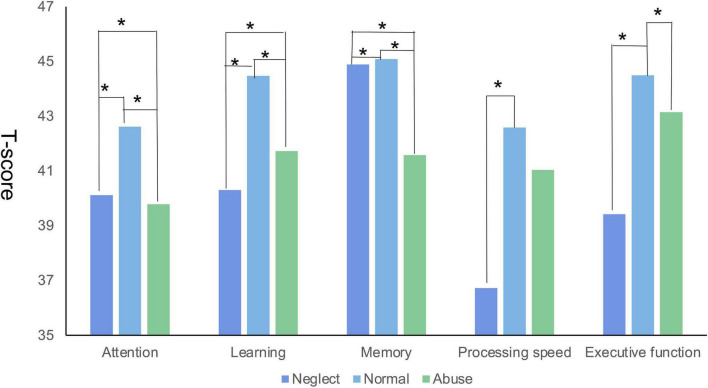
The differences of the T-score in each cognitive domain among different latent classes (*represents *P* < 0.05).

We compared the differences in the T-scores for each cognitive domain and each test among different classes and between two groups, as shown in Table 4. Unexpectedly, the MDD group had worse cognitive function on all dimensions than the healthy group. In general, participants who had a history of maltreatment tended to perform more poorly on the Continuous Performance Test, the Hopkins Verbal Learning Test, the Brief Visuospatial Memory Test, the Digit Symbol Coding Test, and the Stroop Color and Word Test. The results revealed significant differences between the different classes in cognitive functioning in five domains. SNK test results showed that compared to the other two classes, the neglect class had lower cognitive function scores in learning, processing speed, and executive function. The abuse class had the lowest scores in attention and memory (Figure 4).

**TABLE 4:** T-scores of neurocognitive test among three classes and two groups.

	Classes					Groups			
	**Neglect**	**Normal**	**Abuse**	***Z***	***P***	**MDD**	**HC**	***t***	***P***
Attention	40.12 (12.15)	42.62 (11.57)	39.78 (11.12)	3.27	0.0385	41.08 (11.81)	44.95 (10.40)	4.66	<0.001
CPT-IP_2	38.82 (11.96)	42.27 (11.08)	39.89 (11.25)	2.91	0.0551	41.00 (11.36)	44.05 (10.29)	3.59	0.0004
CPT-IP_3	43.00 (12.67)	42.61 (10.83)	40.95 (10.86)	1.18	0.3077	41.41 (11.13)	44.84 (9.81)	4.37	<0.001
CPT-IP_4	41.06 (12.89)	45.05 (10.86)	41.97 (9.53)	4.96	0.0072	43.54 (10.88)	47.05 (10.13)	4.28	<0.001
CPT-IP_mean	40.12 (12.15)	42.62 (11.57)	39.78 (11.12)	3.27	0.0385	41.08 (11.81)	44.95 (10.40)	4.66	<0.001
Learning	40.31 (16.33)	44.47 (11.52)	41.72 (11.92)	4.23	0.0148	42.80 (11.95)	46.75 (10.74)	5.07	<0.001
hvltl	38.24 (16.50)	42.78 (12.00)	40.13 (13.12)	3.85	0.0216	40.98 (12.43)	45.37 (11.47)	5.15	<0.001
bvmtl	42.38 (20.30)	46.16 (15.21)	43.31 (14.71)	2.50	0.0826	44.62 (15.25)	48.13 (15.14)	3.30	0.0010
Memory	44.89 (9.66)	45.08 (10.59)	41.58 (11.99)	5.78	0.0032	43.20 (11.24)	47.86 (9.02)	6.69	<0.001
hvltd	43.63 (10.15)	44.58 (12.14)	41.56 (12.76)	3.45	0.0322	42.54 (12.38)	47.85 (11.01)	6.59	<0.001
bvmtd	46.05 (11.01)	45.64 (12.33)	41.48 (14.40)	6.02	0.0025	43.82 (13.39)	48.00 (10.26)	5.18	<0.001
Processing speed	36.72 (11.88)	42.58 (9.29)	41.03 (7.76)	3.54	0.0296	41.09 (9.48)	45.03 (7.79)	6.29	<0.001
ctt1	34.58 (13.41)	38.45 (11.87)	37.29 (10.73)	1.46	0.2335	37.03 (11.85)	40.93 (11.08)	4.69	<0.001
spword	39.11 (13.37)	45.65 (12.98)	43.98 (11.34)	3.04	0.0483	44.24 (13.05)	47.78 (11.86)	3.78	0.0002
spcolor	41.89 (11.74)	45.98 (12.41)	45.8 (12.96)	0.95	0.3878	44.68 (12.33)	48.71 (12.40)	4.43	<0.001
bacs	32.21 (13.50)	40.06 (13.31)	37.21 (11.75)	5.62	0.0037	37.98 (12.90)	42.99 (13.16)	5.47	<0.001
Executive function	39.42 (10.44)	44.49 (9.18)	43.15 (8.59)	3.32	0.0366	43.07 (9.24)	46.90 (8.35)	5.65	<0.001
ctt2	33.42 (10.79)	39.84 (13.86)	38.65 (12.76)	2.36	0.0945	38.09 (13.39)	42.89 (13.78)	4.95	<0.001
spinterference	42.67 (12.86)	48.86 (12.54)	46.66 (12.52)	3.54	0.0295	47.25 (12.48)	51.23 (12.39)	4.32	<0.001
Animal	43.68 (15.17)	44.95 (11.98)	43.46 (11.47)	0.93	0.3939	43.54 (12.12)	47.41 (11.21)	4.64	<0.001

CPT-IP, the continuous performance test-identical pairs; hvltl, the immediate recall of the Hopkins verbal learning test-revised; bvmtl, the immediate recall of the brief visuospatial memory test-revised; hvltd, the delayed recall T-scores of the Hopkins verbal learning test-revised; bvmtd, the delayed recall T-scores of the brief visuospatial memory test-revised; ctt1, the color trails test I; spword, word task of stroop color-word test; spcolor, color task of stroop color-word test; bacs, digit-symbol coding test; ctt2, the color trails test II; spinterference, stroop color-word test (word interference task); animal, the animal verbal fluency scale.

## 4. Discussion

Clinical trial data covering both multicenter MDD patients and healthy controls were examined in this study. The prevalence of CM was higher in MDD patients (41.59%) than in the healthy control group (31.02%), which was consistent with a previous study (38). The MDD group had lower positive coping style scores and higher negative coping style scores. A meta-analysis showed that coping styles characterized by a self-destructive, avoidant, or impulsive response to a given problem or stressor increased the risk for subsequent depression (39). Furthermore, this study examined multi-type CM’s role in the network relationships between personality dimensions and coping styles. LCA was applied to determine the three latent classes of CM and address the cognitive function differences among them.

### 4.1. Interrelationships between different types of CM, personality dimensions, and individual coping style

The network analysis showed that CM, especially childhood neglect, played an essential role in personality traits and coping styles. Neglect had the highest number and strength of the connections, which meant it might have intermediary effects or be more closely related to all other aspects. The moderating effect of neglect had been found on life satisfaction, depressive symptoms, cognitive functions, treatment outcomes, and suicide risk (40). Social learning theory emphasizes the importance of observing and modeling the behaviors, attitudes, and emotional reactions of others. Children spontaneously imitate their parents to learn reactivity, and disciplinary methods are affected by the family atmosphere (41).

In our study, participants with neglect experience tended to exhibit hyper-reactivity to negative emotions as a result of acquiring negative affect and poor regulation strategies from their parents (42). However, neglect was often ignored in previous studies (43). Physical and sexual abuse is easier to identify and tends to attract more attention from social workers and researchers, even the media (44, 45). Neglect is much more challenging to identify; it is characterized by a lack of care and protection rather than direct explicit abuse. In addition, because children with neglect might be subjected to other forms of abuse simultaneously, the independent impact of neglect was often ignored by researchers. This is particularly concerning because studies suggest that the consequences of childhood neglect might be as severe as those of other types of maltreatment. In the long term, neglect is at least as damaging as physical or sexual abuse, but it receives the least public attention. Surprisingly, sexual abuse did not show significant associations with any of the personality dimensions. Previous findings also showed inconsistent results. This contradiction might be caused by the biases in self-reports of sexual abuse. When answering questions related to sex, there might be problems such as forgetting, denial, misunderstanding, and embarrassment that are likely to lead to the under-reporting of the sexual abuse of children (46). Besides, children’s sex education in China is not universal, which could lead to poor ability to recognize sex abuse.

The differences in network structure between groups might indicate the pathological mechanisms of MDD. EN was directly related to P in the healthy group, and PN was directly related to N. However, this relationship disappeared in the MDD group. The person who could buffer the impact of stressful events such as CM on adult personality traits might not develop MDD in adulthood. Although the etiology and pathogenesis of depression are still unclear, CM is regarded as a critical risk factor for depression. This might partly explain why not all children with CM become depressed. Individuals’ personality traits may significantly influence the association between CM and later-life depression (20).

### 4.2. Latent class of CM

Children who were exposed to one type of maltreatment were often exposed to other types. The results of LCA also showed these strong connections, which were in line with previous literature. Furthermore, the particularly strong connections between physical and emotional CM (both abuse and neglect) confirmed that physical CM was often accompanied by emotional CM. Though the MDD group was divided into three classes and the healthy group was grouped into two classes, the kinds of classifications were still similar. PN was in a dominant position in class 1, accompanied by EN, in both populations. This also demonstrated that the co-occurrence of different types of CM was more common within abuse (i.e., PA and EA) or neglect (i.e., PN and EN) than between abuse and neglect (i.e., PN and EA). The main difference was in Class 3 of the MDD group. EA was in the dominant position, accompanied by PN and PA. Showing that neglect and abuse co-occur more commonly in MDD patients compared to the healthy population. It should be highlighted here that neglect and abuse likely represent the two extreme polarities of CM. Neglect is the most relevant form of maltreatment “by omission,” in which the child is deprived of the basic needs of protection, care, and love from caregivers. On the contrary, abuse represents “by commission”; the caregivers degrade, humiliate, and terrorize their children to show their power or control over them. According to Infurna’s meta-analysis (47), both types may develop a more negative self-model, becoming prone to internalizing symptoms, which may easily foster depression later in life.

### 4.3. Relationship between CM and adult cognitive functioning

This study investigated the relationship between latent classes of CM and adult cognitive functioning. A systematic review was previously conducted evaluating the evidence for an association between maltreatment and cognition in children under 12 years. However, there has been little research on adult cognitive function. Significant differences were found in five domains among the different classes (i.e., attention, learning, memory, processing speed, and executive function). CM (both abuse and neglect) was associated with the development of cognitive functioning. There are two possible explanations for this. One is that children with maltreatment could have potential differences in brain anatomy or patterns of disrupted cognitive functioning compared to those without (48). The other might be the equivocal causation between CM and cognitive function (49).

Furthermore, the two core dimensions of CM—abuse and neglect—might influence different dimensions of cognitive function. Our results showed that the neglect class performed more poorly in learning, processing speed, and executive function, while the abuse class performed more poorly in attention and memory. This might be because different CM experiences were related to the development of two underlying neural processes of cognition. Specifically, neglect was a more prominent predictor for developmental changes in insula-dACC activation during risk processing, while abuse was a more prominent predictor for developmental changes in frontoparietal activation during cognitive control (50). However, the association between maltreatment and adulthood cognition is a complex and pendent problem. Notably, when considering causality, the evidence from cross-sectional studies is weak, as the direction of causality could be from maltreatment to cognitive problems or vice versa (51). Danese et al. (49) found that even though there was impairment in cognitive functioning among those exposed to CM, this impairment was explained mainly by cognitive difficulties that pre-dated CM exposure and confounding genetic and environmental factors. As a cross-sectional study, the results here could only describe the difference. Longitudinal studies are needed in the future to explore the mechanics behind it.

## 5. Limitations

There are some limitations of this study. First, our measures were self-report questionnaires, which raised the potential problem of response bias, as participants might underreport past CM. CM might also be underestimated because of its stigma, especially for sexual abuse. Respondents might be ashamed to share it with the investigators. However, this is currently an unavoidable problem. Studies that have linked self-reports to official statistics for child protection provide direct evidence of underreporting to agencies. One study reported evidence of contact with child protection services in only 5% of children who were physically abused and 8% of those who were sexually abused. The co-occurrence is underestimated by official reports because the recording of more than one type of maltreatment is often discouraged by child protection agencies (3). Second, as the MDD and healthy groups were not matched by age, gender, and educational level, there were significant differences in the social-demographic characteristics of the MDD and control groups. The control group was younger and had a higher educational level and employment rate, which might influence the relationships among CM, personality traits, and coping style. It is important to be cautious in interpreting these results. Third, the cross-sectional evaluation limits the results. With these data, we are not able to obtain the longitudinal relationship and cannot determine a causal relationship between CM and cognitive impairment.

## 6. Future research

Child maltreatment is common, and for many, it is a far-reaching condition, with adverse outcomes throughout childhood and into adulthood. The high burden and severe and long-lasting consequences of CM warrant increased investment in preventive and therapeutic strategies from early childhood. More attention needs to be paid to neglected children. There is mounting evidence that the effects of childhood neglect can be as damaging—or perhaps even more damaging—to a child as physical or sexual abuse. More research is needed into the characteristics of responses by communities, families, and services that help with healthy development rather than exacerbating the child’s problems, such as improving the understanding of how children are victimized at different stages of development.

## Data availability statement

The original contributions presented in this study are included in this article/Supplementary material, further inquiries can be directed to the corresponding authors.

## Ethics statement

The studies involving human participants were reviewed and approved by the Ethic Committee of Peking University Sixth Hospital. The patients/participants provided their written informed consent to participate in this study.

## Author contributions

XW, SL, and YX originally designed the idea of the study, had responsible for obtaining funding, and contributed to the study design and development of study instruments. XL, JZ, XD, JXL, WZ, and JL collected the data. XW and SL undertook data cleaning, checking, and coding, analyzed the data for the study, and wrote the initial draft. XL, WZ, and XD contributed to the amendment of the manuscript and suggestions for data analysis. YX conceived the idea for this manuscript, supervised and checked the analysis, and wrote the final draft. All authors contributed to the interpretation of data and the approval of the final report.
