# Supplementary material for: Effect of childhood maltreatment on cognitive function and its relationship with personality development and social coping style in major depression disorder patients: A latent class model and network analysis
Source: Front Psychiatry. 2023 Jan 20;14:748857. doi: 10.3389/fpsyt.2023.748857 (PMC9895412; doi:10.3389/fpsyt.2023.748857)
Supplement: Supplementary file 1 [file Data_Sheet_1.docx]

Supplementary Material

1. The participants were recruited from 9 clinical sites at 9 top tertiary hospitals (7 within academic settings and 2 in clinical practices) located in 6 cities. The number of cases recruited by each clinical site is shown in Supplementary Table S1.

| Table S1 The sample size of each clinical site | | |
| --- | --- | --- |
| Clinical site | MDD  (n) | Healthy control  (n) |
| The First Hospital of China Medical University | 145 | 47 |
| Dalian Mental Health Center | 63 | 41 |
| Tianjin Anding Hospital | 177 | 35 |
| Tianjin Medical University General Hospital | 47 | 31 |
| The First Hospital of Shanxi Medical University | 151 | 38 |
| The First Hospital of Hebei Medical University | 101 | 38 |
| Peking Union Medical College Hospital | 170 | 59 |
| Beijing Anding Hospital | 112 | 151 |
| Peking University Sixth Hospital | 164 | 59 |
| Total | 1130 | 499 |

1. The details of inclusion and exclusion criteria for each group are listed in Supplementary Table S2.

| Table S2 The inclusion and exclusion criteria for MDD patients and healthy controls, respectively | | |
| --- | --- | --- |
| Study group | Inclusion criteria | Exclusion criteria |
| MDD  patients | 1. age between 18 and 55 years at the time of enrollment; 2. diagnosis of MDD based on the Chinese Version of MINI according to DSM-IV TR; 3. first-episode or relapsed; 4. having the ability of reading and writing to complete the questionnaire and psychological assessment; 5. providing written confirmation of informed consent. | 1. lifetime or current diagnosis of other psychotic disorder, alcohol/substances dependence, or cognitive impairment; 2. severe somatic diseases, such as severe cardio-cerebral vascular diseases, respiratory diseases, liver diseases, kidney diseases, or malignant tumors; 3. not signed the informed consent; 4. been engaging in other studies |
| Healthy  controls | 1. age between 18 and 55 years at the time of enrollment; 2. providing written confirmation of informed consent prior to engaging the study. | 1. lifetime or current diagnosis of any mental diseases; 2. severe somatic diseases, such as severe cardio-cerebral vascular diseases, respiratory diseases, liver diseases, kidney diseases, or malignant tumors; 3. not signed the informed consent; 4. been engaging other studies. |

1. The details of model fit information for latent class analysis are listed in Supplementary Table S3.

| Table S3 Model fit information for latent class analysis | | | | | | |
| --- | --- | --- | --- | --- | --- | --- |
|  | AIC | BIC | SS-BIC | Entropy | LMR-LRT | BLRT |
| **MDD** |  |  |  |  |  |  |
| 2-class | 3588.71 | 3644.04 | 3609.10 | 0.809 | *P*<0.001 | *P*<0.001 |
| 3-class | 3548.66 | 3634.17 | 3580.17 | 0.917 | *P*=0.0039 | *P*<0.001 |
| 4-class | 3551.35 | 3667.04 | 3593.98 | 0.945 | *P*=0.0255 | 0.1364 |
| **HC** |  |  |  |  |  |  |
| 2-class | 1069.43 | 1115.77 | 1080.85 | 0.92 | *P*<0.001 | *P*<0.001 |
| 3-class | 1071.101 | 1142.716 | 1088.757 | 0.998 | *P*=0.0039 | *P*<0.001 |
| 4-class | 1074.317 | 1171.207 | 1098.204 | 0.999 | *P*<0.001 | *P*=0.03 |

1. Network Analysis

Stability and robustness of the edge weight estimates by drawing bootstrapped 95% confidence intervals (CIs); if 1000 different subsamples within the larger sample have estimates that do not change too much (i.e., narrower CIs), it is likely that estimates are representative for current sample (Figure S1 and S2).


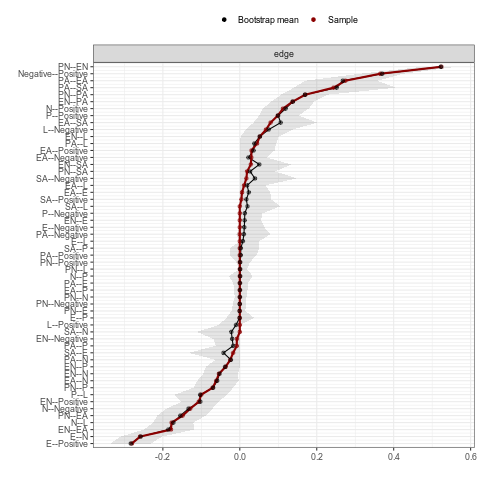


**Figure S1.** Stability of the edge weights in MDD group. (Edge weights (thick line) and the 95% CIs around these edge weights (grey bars). The edges are arranged such that the one with the highest edge-weight is at the top and the lowest edge-weight at the bottom. Ideally, narrower CIs indicate a more stable and robust estimation of the edge weights.)


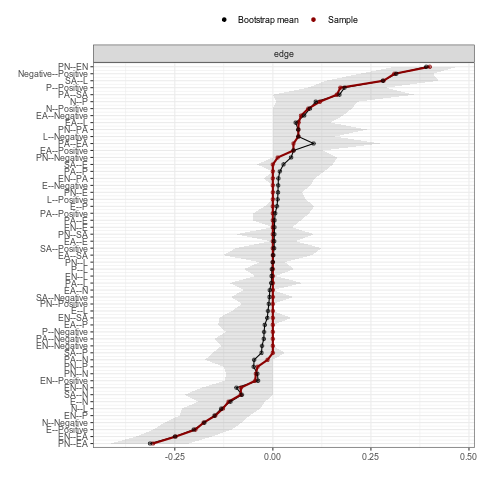


**Figure S2.** Stability of the edge weights in healthy group. (Edge weights (thick line) and the 95% CIs around these edge weights (grey bars). The edges are arranged such that the one with the highest edge-weight is at the top and the lowest edge-weight at the bottom. Ideally, narrower CIs indicate a more stable and robust estimation of the edge weights.)
